# The impact of job stress on job satisfaction and turnover intentions among bank employees during the COVID-19 pandemic

**DOI:** 10.3389/fpsyg.2024.1482968

**Published:** 2024-10-25

**Authors:** Mei-Hui Lin, Ya-Hui Yen, Tsai-Fu Chuang, Ping-Sen Yang, Ming-Da Chuang

**Affiliations:** ^1^Department of Public Finance, Feng Chia University, Taichung City, Taiwan; ^2^Department of Nursing, National Chi Nan University, Nantou County, Taiwan; ^3^Department of Civil Engineering, Feng Chia University, Taichung City, Taiwan; ^4^Department of Accounting and Information Management, Da-Yeh University, Changhua County, Taiwan; ^5^Department of Computer Science and Engineering, National Sun Yat-sen University, Kaohsiung City, Taiwan

**Keywords:** job stress, job satisfaction, turnover intention, health, bank employee

## Abstract

**Objective:**

The main objective of this study is to explore the relationships among job stress, job satisfaction, and turnover intentions among bank employees during the COVID-19 pandemic, with a focus on variations across different demographic characteristics.

**Methods:**

A cross-sectional design was employed, and data were collected from 501 bank employees using the Job Stress Questionnaire (JSQ), the Simplified Minnesota Satisfaction Questionnaire (MSQ), and the Intention to Leave Scale (ILS). Descriptive statistics, t-tests, ANOVA, Pearson correlation, and multiple regression analyses were used to test the research hypotheses.

**Results:**

The findings show that job stress is significantly negatively correlated with job satisfaction and positively correlated with turnover intention. Interpersonal relationship stress emerged as the strongest predictor of turnover intention, while job autonomy stress significantly influenced job satisfaction. Demographic factors, including age, income, and education level, moderated these relationships, with younger, higher-income, and more educated employees reporting lower stress and higher satisfaction. Employees with dependents reported higher stress levels, lower job satisfaction, and greater turnover intentions compared to those without dependents.

**Conclusion:**

This study underscores the importance of managing workplace stress and enhancing job satisfaction to reduce turnover intention, particularly during the COVID-19 pandemic. Interventions focused on improving interpersonal relationships and providing targeted support for older and lower-income employees are recommended to mitigate stress and improve retention rates.

## 1 Introduction

Job stress, characterized by workplace-induced anxiety and depression, can lead to job dissatisfaction, absenteeism, reduced working hours, and decreased productivity, ultimately impairing an organization’s normal operations (Armitage and Nellums, 2020; Navarro-Prados et al., 2024; Singh et al., 2019; Webster et al., 2010). Job dissatisfaction is a common outcome of work stress, along with symptoms such as depression, anxiety, boredom, frustration, isolation, and hostility. Reduced job satisfaction can lead to turnover due to diminished organizational commitment. Work stress impacts both personal and professional life, imposing significant costs on organizations, making it a critical issue (Singh et al., 2019). Job stress encompasses various symptoms across physiological, psychological, and behavioral aspects. When workplace expectations exceed an individual’s capacity and resources, poor health conditions and even injury can occur (Singh et al., 2019).

In contrast, job satisfaction reflects an individual’s attitude toward their work on the basis of personal perceptions. Job satisfaction enhances individual productivity and organizational commitment, promoting personal well-being and life satisfaction (Sabbarwal et al., 2017). Previous studies have demonstrated that job satisfaction serves as a cornerstone for management policies aimed at enhancing organizational productivity and efficiency (Bottonari et al., 2007). Job satisfaction impacts both individual members and overall organizational performance, whereas stress is a critical factor experienced by workers in numerous professions, directly affecting job satisfaction (Friganovic et al., 2019). Managers should prioritize job satisfaction, as dissatisfied individuals are more likely to delay or be absent from work, whereas satisfied individuals are more efficient and committed (Sabbarwal et al., 2017).

A significant inverse relationship exists between work stress and job satisfaction, with higher levels of work stress being associated with lower job satisfaction (Ahsan et al., 2009). Previous research has consistently shown that job stress predicts job satisfaction; higher work stress leads to lower job satisfaction (Ahsan et al., 2009; Cummins, 1990; Fletcher and Payne, 1980; Klassen et al., 2009; Koslowsky et al., 1995; Landsbergis, 1988; Stamps and Piedmonte, 1986; Vinokur-Kaplan, 1990; Yang et al., 2018). For example, a 2021 study on delivery workers revealed a significant negative correlation between work stress and job satisfaction (*r* = −0.266, *p* < 0.01) (Xie et al., 2021). In Taiwan, studies have shown that work stress negatively affects job satisfaction (Chen and Huang, 2019; Wu and Chen, 2022; Zhang and Ru, 2021) and that work stress has a direct negative effect on teachers’ job satisfaction (Wu and Chen, 2022).

The COVID-19 pandemic has caused substantial damage to governments, economies, and businesses globally. In response to the pandemic, numerous governments have implemented various economic policies to protect the real economic sector from the adverse effects of the pandemic. Despite these efforts, many economies remain vulnerable to the ongoing impacts of COVID-19 (Obuobisa-Darko and Sokro, 2023; Takyi et al., 2023). The pandemic has particularly intensified challenges in high-stress work environments, contributing to increased employee dissatisfaction and turnover intention. For example, during the pandemic, stressful work conditions significantly heightened employee dissatisfaction and turnover intentions. Research has shown that enhancing job satisfaction, particularly among nurses, can increase their organizational commitment and reduce turnover intentions (Said and El-Shafei, 2021). Studies have shown that greater psychological workload, lower perceived organizational justice, and greater turnover intention are significantly related to work stress (Chen and Huang, 2019; Feng et al., 2020; Hong, 2021; Hoboubi et al., 2017; Su and Zhu, 2018; Tsai and Lin, 2018; Wang and Huang, 2018; Wu et al., 2022; Xu, 2021; Yang and Lu, 2017; Zhang and Ru, 2021). Research indicates that job satisfaction negatively affects turnover intention (Hong, 2021; Li and Hong, 2022; Lin et al., 2022; Su and Zhu, 2018; Tsai and Lin, 2018; Tsai and Wu, 2022; Wu, 2017; Wu et al., 2022; Xu et al., 2022; Xu, 2021; Zhang and Ru, 2021).

Although much of the literature has focused on healthcare workers, educators, students, and couriers—who were directly impacted by the pandemic owing to their roles in frontline healthcare and educational settings—there has been a relative paucity of research examining the banking sector. Despite this, bank employees faced a unique set of challenges during the pandemic, including increased workloads, accelerated technological changes, heightened customer demands, and stringent compliance requirements. These factors significantly intensify both psychological and physiological stress among bank employees, negatively affecting their job satisfaction and increasing their turnover intention.

This lack of research on bank employees represents a critical gap in our understanding of how the COVID-19 pandemic has affected various professional groups. The increased job stress and decreased job satisfaction among bank employees are pressing concerns, as they directly influence turnover intention, posing substantial risks to organizational stability and performance. High turnover rates can lead to increased recruitment and training costs while also undermining overall organizational effectiveness. As core institutions within the economic system, banks play a vital role in societal stability, and the well-being of their employees is crucial not only to the organization but also to the broader economy. Given these considerations, studying job stress, job satisfaction, and turnover intentions among bank employees during the COVID-19 pandemic is essential. This research aims to fill the existing gap by providing empirical data that can help management develop effective strategies to reduce job stress, increase job satisfaction, and ultimately decrease turnover rates.

On the basis of these premises, the primary aim of this study is to explore and analyze the relationships among job stress, job satisfaction, and turnover intention. The specific objectives are as follows:

1. To examine the variances in job stress, job satisfaction, and turnover intention across individuals from diverse demographic backgrounds.
2. To analyze the interrelationships among job stress, job satisfaction, and turnover intention within a sample of bank employees.
3. To investigate the impact of job stress on job satisfaction among bank employees.
4. To assess the predictive influence of job stress and job satisfaction on turnover intention among bank employees.

## 2 Methods

### 2.1 Research framework

**Figure 1:**
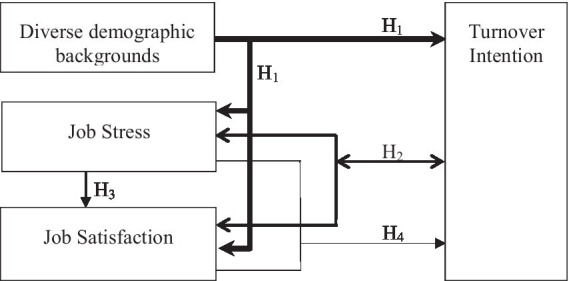
Research framework.

The research framework is illustrated in Figure 1. This diagram delineates the relationships among the study variables. Job stress and job satisfaction are the primary independent variables influencing the study, whereas turnover intention serves as the dependent outcome variable. Additionally, diverse demographic backgrounds are considered potential moderating factors that may influence these relationships.

The research hypotheses of this study are as follows:

1. Research Hypothesis 1 (H_1_): There are significant variances in job stress, job satisfaction, and turnover intention across individuals from diverse demographic backgrounds.
2. Research Hypothesis 2 (H_2_): There is a significant correlation among job stress, job satisfaction, and turnover intention.
3. Research Hypothesis 3 (H_3_): Job stress significantly influences employees’ job satisfaction.
4. Research Hypothesis 4 (H_4_): Job stress and job satisfaction significantly influence employees’ turnover intention.

### 2.2 Research instrument

This study employs three attitudinal measurement instruments: the simplified job stress questionnaire (JSQ) from Taiwan’s Ministry of Labor (2020), the Minnesota Satisfaction Questionnaire (MSQ) (University of Minnesota, 2020), and the Intention to Leave Scale (ILS) developed by Scott et al. (1999). The JSQ consists of 23 items, the short-form MSQ contains 20 items, and the ILS includes 4 items. The Job Stress Questionnaire (JSQ) and the Minnesota Satisfaction Questionnaire (MSQ) were utilized in their original forms.

The JSQ demonstrated strong internal consistency, with an overall Cronbach’s *α* of 0.973 and a cumulative variance explained of 84.274% (KMO = 0.917, *p* < 0.001). The exploratory factor analysis (EFA) of the Job Stress questionnaire identified a three-factor structure. The rotated sums of squared loadings indicate that the first factor accounts for 22.165% of the total variance, the second factor explains an additional 21.437%, and the third factor contributes 5.506%. Collectively, these three factors explain 49.108% of the total variance, suggesting that the identified factors capture a substantial portion of the variability in job stress among the participants. It consists of three dimensions, namely, job autonomy (Hackman and Oldham, 1975), workload, and interpersonal relationships at the workplace (Spector and Jex, 1998), along with a single item to assess overall job stress. The Cronbach’s α values for the three dimensions were 0.881 (variance explained = 51.568%), 0.870 (variance explained = 67.97%), and 0.998 (variance explained = 99.7%), respectively, with all *p* values being statistically significant (*p* < 0.001) and eigenvalues exceeding 1.

Based on the confirmatory factor analysis (CFA) conducted for the Job Stress instrument, both the one-factor and three-factor models were evaluated using several fit indices. The results indicate that the *p*-value of the Chi-square test for both models is 0, with the Chi-square/df ratio slightly lower for the three-factor model (4.9) compared to the one-factor model (5.1), suggesting a marginally better fit for the three-factor model. The RMSEA (Root Mean Square Error of Approximation) values are higher than the recommended threshold of 0.08 for both models, with the one-factor model showing an RMSEA of 0.090941 and the three-factor model slightly lower at 0.088535, indicating that neither model fits the data exceptionally well according to this index.

However, the CFI (Comparative Fit Index) values are above the threshold of 0.90 for both models, with the one-factor model at 0.983901 and the three-factor model at 0.984742, indicating a good fit for both models according to this index. Similarly, the TLI (Tucker-Lewis Index) values for both models are above the acceptable threshold of 0.90, with the one-factor model at 0.992351 and the three-factor model at 0.992756, again indicating a strong model fit. Finally, the SRMR (Standardized Root Mean Square Residual) values exceed the recommended threshold of 0.08 for both models, with the one-factor model at 0.09976 and the three-factor model slightly lower at 0.096279. Although the SRMR values suggest that neither model achieves an optimal fit, the three-factor model consistently demonstrates marginally better performance across all indices.

These results provide evidence that, while neither model achieves perfect fit across all indices, the three-factor model offers a slightly better fit compared to the one-factor model, supporting its use in subsequent analyses.

The MSQ, in its abbreviated version, measures job satisfaction by evaluating intrinsic and extrinsic factors. This classification is grounded in Herzberg’s two-factor theory, which asserts that intrinsic motivators and extrinsic hygiene factors play distinct roles in shaping an individual’s job satisfaction (Sonnenschein et al., 2022). Intrinsic and extrinsic factors are commonly assessed in job satisfaction evaluations, as posited by Herzberg’s two-factor theory. Intrinsic factors, related to the nature of the job and internal fulfillment, include elements such as job challenge, significance, achievement, personal growth opportunities, and self-fulfillment. Conversely, extrinsic factors pertain to external conditions and the work environment, such as salary and benefits, work conditions, interpersonal relationships, job security, and organizational policies. These dimensions influence overall job satisfaction by addressing different aspects of the work experience (Wang et al., 2021). The MSQ also demonstrated acceptable reliability, with an overall Cronbach’s *α* of 0.816 and a cumulative variance explained of 61.116% (KMO = 0.897, *p* < 0.001). The MSQ measures two dimensions, intrinsic and extrinsic factors, with Cronbach’s α values of 0.882 (variance explained = 41.260%) and 0.805 (variance explained = 46.237%), respectively. Both dimensions had eigenvalues greater than 1, and the results were statistically significant (*p* < 0.001).

Additionally, the intention-to-leave scale (ILS), which was originally developed by Scott et al. (1999) through the combination of items from questionnaires by Mobley (1977) and Hom et al. (1984), was modified in this study by incorporating four positively worded items. This adaptation aimed to enhance respondent comprehension and facilitate completion. The original semantics of the ILS items were revised into positive statements to improve clarity and ease of response for participants.

All three research questionnaires—the Job Stress Questionnaire (JSQ), the Minnesota Satisfaction Questionnaire (MSQ), and the Intention to Leave Scale (ILS)—utilize a 5-point Likert scale (1 = strongly disagree, 2 = disagree, 3 = neutral, 4 = agree, 5 = strongly agree). In the JSQ, higher scores originally indicated lower levels of stress. However, to ensure consistency and facilitate accurate analysis, these scores were reversed so that higher scores uniformly represented greater levels of stress. This adjustment included four items that were initially reverse-coded and were also rescored to align with this approach. In the satisfaction and turnover intention questionnaires, a higher score indicates greater satisfaction and turnover intention, respectively, with no reverse-coded items present in these questionnaires. Moreover, in our study, we made minimal modifications to the questionnaires specifically focusing on language adjustments to better align with our research context and participant demographics. We have ensured that the scale’s integrity and validity were preserved throughout the process.

### 2.3 Samples and data collection

The research sample for this study was drawn from banks in central Taiwan, with data collection conducted via Google Forms and electronic questionnaires distributed via Line and email. A total of 551 questionnaires were distributed via convenience sampling, and after 50 incomplete responses were excluded, 501 valid responses remained, resulting in a valid response rate of approximately 91%. Participation was entirely voluntary, and stringent anonymization procedures were not needed, as participants were not asked to disclose their names or identify specific service institutions.

As for the exclusion of 50 questionnaires, they were excluded because they did not meet the completion criteria for analysis. Specifically, the demographic background variables were intentionally set as optional in the questionnaire to respect respondents’ autonomy, allowing them the freedom to choose whether to provide such information. However, 50 questionnaires were returned with incomplete demographic data, which led to their exclusion. This decision was made to ensure that the dataset used for analysis was both complete and robust, preserving the integrity of the research findings.

The foundational information about the research samples is briefly presented below, with comprehensive and detailed distributional data provided in Table 1.

**Table 1:** Frequency distribution of demographic variables.

Variable	Category	Number of people	Percentage (%)
Gender	Male	214	42.7%
	Female	287	57.3%
Age	30 and below	30	6.0%
	31–40	231	46.1%
	41–50	187	37.3%
	51 and above	53	10.6%
Monthly income (TW$)	30,000 and below	93	18.6%
	30,001–35,000	191	38.1%
	35,001–50,000	181	36.1%
	50,001 and above	36	7.3%
Education level	High school or below	66	13.2%
	College (including incomplete)	238	47.5%
	Master’s or above (including incomplete and ongoing)	197	39.3%
Job position	Nonsupervisory	286	57.2%
	Supervisory	215	42.8%
Marital status	Married	116	23.2%
	Unmarried	385	76.8%
Dependents	Yes	391	78.0%
	No	110	22.0%

*N = 501.*

The sample consisted of 42.7% male and 57.3% female respondents, indicating a greater number of female participants. The largest age group was 31–40 years old, accounting for 46.1% of the sample, followed by the 41–50 age group at 37.3%, and those 30 years old and younger made up 6.0% of the sample.

In terms of monthly income, the most common range was NT$30,001–35,000, accounting for 38.1% of the respondents, followed by NT$35,001–50,000, accounting for 36.1%, and those earning NT$50,001 and above, accounting for 7.3%. With respect to education level, 47.5% of the respondents had a college education (including those who did not complete their degree), followed by those with a master’s degree or higher (including those currently studying or who did not complete their degree) at 39.3%, and those with a high school education or less at 13.2%.

Nonsupervisory positions accounted for 57.2% of the sample, whereas supervisory positions accounted for 42.8%. The majority of the respondents were unmarried (76.8%), with 23.2% being married. Additionally, respondents without dependents composed 22% of the sample, whereas those with dependents made up 78% of the sample.

### 2.4 Procedure

The procedure for this study was carefully designed to ensure a thorough and reliable data collection process. The data collection period for this study spanned from May 1, 2022, to September 30, 2022. Participants were recruited from banks located in central Taiwan, specifically in Miaoli County, Taichung City, Changhua County, Yunlin County, and Nantou County. The selection of banks was guided by official data from Central Bank of Republic of China (2022), which identified 625 branches of domestic banks and 4 branches of foreign banks operating in the central region during 2022. It is important to note that this study did not include other financial institutions such as credit cooperatives, credit departments of farmers’ and fishermen’s associations, life insurance companies, property insurance companies, and financial holding companies. Participants were recruited from banks in central Taiwan, specifically from Miaoli County, Taichung City, Changhua County, Yunlin County, and Nantou County. To extend the survey’s reach, bank employees, faculty members from educational institutions, and members of various organizations and civil associations were enlisted to help distribute the questionnaires. Additionally, friends and family were encouraged to invite eligible participants. The study’s purpose was clearly communicated, and participants were invited to participate voluntarily.

Eligible participants were required to be current bank employees in central Taiwan. Informed consent was obtained at the start of the questionnaire. Those who did not provide consent or did not meet the inclusion criteria were excluded from the study. The participants were also instructed to carefully read the informed consent information before providing demographic details and completing a 47-item questionnaire, which took approximately 30–35 min. The survey was conducted anonymously to protect participant confidentiality.

To ensure the reliability and validity of the data collection, the survey was pretested with a small sample of bank employees. Feedback from the pretest was used to refine the questionnaire, enhancing its clarity and effectiveness. The emphasis on anonymity was intended to encourage honest and accurate responses.

Moreover, the survey conducted in this study was in full compliance with the principles of the Declaration of Helsinki. Informed consent was obtained from all participants. At the beginning of the survey, the participants were provided with detailed information about the study and consented to participate. They then provided their demographic information before completing the questionnaire. The survey was administered anonymously, ensuring that no personal identifiers could be traced from the data.

### 2.5 Data analysis

For the data analysis, IBM SPSS Statistics version 24 was used to perform both descriptive and inferential statistical analyses. Descriptive statistics, including percentages, were used to present the distribution of the demographic data. The current levels of job stress, job satisfaction, and turnover intention within the sample were summarized via means and standard deviations. To investigate potential differences in job stress, job satisfaction, and turnover intention across diverse demographic groups (H_1_), ANOVA or t tests were applied, depending on the number of groups compared. Pearson’s product–moment correlation analysis was conducted to examine the relationships between job stress, job satisfaction, and turnover intention (H_2_). Multiple regression analysis was subsequently performed to assess the predictive power of job stress for job satisfaction and the combined predictive power of job stress and job satisfaction for turnover intention (H_3_ and H_4_). These statistical methods were selected to ensure a comprehensive and accurate analysis, align with the research hypotheses and support the robustness and reliability of the findings.

## 3 Results

### 3.1 Current status of job stress, job satisfaction, and turnover intention

Table 2 presents the descriptive statistics for job stress (JSQ), job satisfaction (MSQ), and turnover intention (TI) among the 501 participants. The mean scores for job stress were 1.818 (SD = 0.559) for job autonomy stress, 2.005 (SD = 0.678) for workload stress, and 2.098 (SD = 0.863) for work-related stress, with an overall mean score of 1.968 (SD = 0.664). For job satisfaction, the mean scores were 3.561 (SD = 0.303) for intrinsic job satisfaction and 3.987 (SD = 0.398) for extrinsic job satisfaction, with an overall mean score of 3.371 (SD = 0.303). The overall mean score for turnover intention was 2.044 (SD = 0.768). These results suggest that the participants experienced relatively low job stress, high job satisfaction, and relatively low turnover intention.

**Table 2:** Summary of descriptive statistics for job stress, job satisfaction, and turnover intention.

Dimension	Mean (M)	Standard deviation (SD)	Standard error
Job stress (JSQ)			
Job autonomy stress	1.818	0.559	0.025
Workload stress	2.005	0.678	0.030
Work relationships stress	2.098	0.863	0.039
The overall average of job stress	1.968	0.664	0.030
Job satisfaction (MSQ)			
Intrinsic job satisfaction	3.561	0.303	0.014
Extrinsic job satisfaction	3.987	0.398	0.018
The overall average of job satisfaction	3.731	0.303	0.014
Turnover intention (TI)			
The overall average of turnover intention	2.044	0.768	0.034

*N = 501.*

### 3.2 Results of demographic differences

#### 3.2.1 Gender

An independent samples *t* test was conducted to examine gender differences in job stress, job satisfaction, and turnover intention, with the results summarized in Table 3. No significant gender differences were found in job autonomy stress (*t*(499) = 1.840, *p* = 0.066), workload stress (*t*(499) = 0.574, *p* = 0.566), work relationship stress (*t*(499) = 0.202, *p* = 0.840), or the overall average job stress (*t*(499) = 0.835, *p* = 0.404). Similarly, no significant gender differences were observed in intrinsic job satisfaction (*t*(499) = −0.548, *p* = 0.584), extrinsic job satisfaction (*t*(499) = −0.172, *p* = 0.863), or the overall average job satisfaction (*t*(499) = −0.420, *p* = 0.675). Additionally, there was no significant gender difference in turnover intention (*t*(499) = 0.282, *p* = 0.778). These results suggest that there are no significant gender differences in job stress, job satisfaction, or turnover intention among the participants in this study.

**Table 3:** Independent samples *t* test results for gender differences in job stress, job satisfaction, and turnover intention.

Dimension	Gender	*N*	Mean (M)	Standard deviation (SD)	*t*	*df*	Sig. (2-tailed)	95% CI lower bound	95% CI upper bound	Cohen’s d
WSQ										
Job autonomy stress	Female	287	1.858	0.558	1.840	499	0.066	−0.006	0.192	0.166
	Male	214	1.765	0.558						
Workload stress	Female	287	2.020	0.680	0.574	499	0.566	−0.085	0.156	0.052
	Male	214	1.985	0.676						
Work relationships stress	Female	287	2.105	0.867	0.202	499	0.840	−0.138	0.169	0.019
	Male	214	2.089	0.859						
The overall average of job stress	Female	287	1.990	0.665	0.835	499	0.404	−0.068	0.168	0.077
	Male	214	1.939	0.663						
MSQ										
Intrinsic job satisfaction	Female	287	3.554	0.306	−0.548	499	0.584	−0.069	0.039	−0.049
	Male	214	3.569	0.301						
Extrinsic job satisfaction	Female	287	3.984	0.387	−0.172	499	0.863	−0.077	0.064	−0.015
	Male	214	3.990	0.412						
The overall average of job satisfaction	Female	287	3.726	0.299	−0.420	499	0.675	−0.065	0.042	−0.040
	Male	214	3.738	0.308						
TI										
The overall average of turnover intention	Female	287	2.052	0.776	0.282	499	0.778	−0.117	0.156	0.025
	Male	214	2.033	0.759						

*N = 501.*

#### 3.2.2 Age

One-way ANOVA revealed significant age-related differences in job stress, job satisfaction, and turnover intention, as detailed in Table 4. Significant differences were observed across age groups for job autonomy stress (*F*(3, 497) = 19.110, *p* < 0.001), workload stress (*F*(3, 497) = 10.893, *p* < 0.001), work relationship stress (*F*(3, 497) = 6.842, *p* < 0.001), and overall job stress (*F*(3, 497) = 12.209, *p* < 0.001), with participants aged over 50 years reporting the highest levels of job stress across all dimensions.

**Table 4:** One-way ANOVA results for age differences in job stress, job satisfaction, and turnover intention.

Dimension	Age group	*N*	Mean (M)	Standard deviation (SD)	95% CI lower bound	95% CI upper bound	*F*	*p* value	*df*	Scheffé *post hoc* test
Job autonomy stress	≤30	30	1.293	0.199	1.218	1.367	19.110^***^	0.000	(3,497)	4. > 3. > 2. > 1.
	31–40	231	1.796	0.560	1.723	1.868				
	41–50	187	1.821	0.540	1.743	1.899				
	>50	53	2.201	0.498	2.064	2.338				
	Total	501	1.818	0.559	1.769	1.867	Partial Eta squared = 0.103			
Workload stress	≤30	30	1.520	0.366	1.383	1.657	10.893^***^	0.000	(3,497)	4. > 3. > 2. > 1.
	31–40	231	1.990	0.680	1.901	2.078				
	41–50	187	1.999	0.657	1.904	2.094				
	>50	53	2.370	0.691	2.179	2.560				
	Total	501	2.005	0.678	1.946	2.065	Partial Eta squared = 0.062			
Works Relationships stress	≤30	30	1.633	0.556	1.426	1.841	6.842^***^	0.000	(3,497)	4. > 2. > 3. > 1.
	31–40	231	2.082	0.873	1.969	2.195				
	41–50	187	2.080	0.842	1.959	2.202				
	>50	53	2.491	0.891	2.245	2.736				
	Total	501	2.098	0.863	2.022	2.174	Partial Eta squared = 0.040			
The overall average of job stress	≤30	30	1.475	0.345	1.347	1.604	12.209^***^	0.000	(3,497)	4. > 3. > 2. > 1.
	31–40	231	1.950	0.670	1.863	2.037				
	41–50	187	1.961	0.641	1.869	2.054				
	>50	53	2.351	0.657	2.170	2.532				
	Total	501	1.968	0.664	1.910	2.026	Partial Eta squared = 0.069			
Intrinsic job satisfaction	≤30	30	3.733	0.136	3.683	3.784	11.905^***^	0.000	(3,497)	1. > 4. > 3. > 2.
	31–40	231	3.557	0.314	3.517	3.598				
	41–50	187	3.592	0.283	3.551	3.633				
	>50	53	3.366	0.304	3.283	3.450				
	Total	501	3.561	0.303	3.534	3.587	Partial Eta squared = 0.067			
Extrinsic job satisfaction	≤30	30	4.404	0.153	4.347	4.461	22.211^***^	0.000	(3,497)	1. > 3. > 2. > 4.
	31–40	231	3.976	0.418	3.922	4.030				
	41–50	187	4.010	0.350	3.960	4.061				
	>50	53	3.712	0.343	3.618	3.807				
	Total	501	3.987	0.398	3.952	4.021	Partial Eta squared = 0.118			
The overall average of job satisfaction	≤30	30	4.002	0.104	3.963	4.040	20.643^***^	0.000	(3,497)	1. > 3. > 2. > 4.
	31–40	231	3.725	0.320	3.683	3.766				
	41–50	187	3.759	0.263	3.721	3.797				
	>50	53	3.505	0.276	3.429	3.581				
	Total	501	3.731	0.303	3.704	3.758	Partial Eta squared = 0.111			
The overall average of turnover intention	≤30	30	1.633	0.556	1.426	1.841	6.863^***^	0.000	(3,497)	4. > 3. > 2. > 1.
	31–40	231	2.026	0.774	1.926	2.126				
	41–50	187	2.032	0.754	1.923	2.141				
	>50	53	2.396	0.768	2.185	2.608				
	Total	501	2.044	0.768	1.976	2.111	Partial Eta squared = 0.040			

*N* = 501; ^***^p < 0.001 Sig. (2-tailed).

Age groups also differed significantly in intrinsic job satisfaction (*F*(3, 497) = 11.905, *p* < 0.001), extrinsic job satisfaction (*F*(3, 497) = 22.211, *p* < 0.001), and overall job satisfaction (*F*(3, 497) = 20.643, *p* < 0.001), with younger participants (under 30) reporting the highest levels of job satisfaction across all dimensions. Turnover intention also varied significantly by age (*F*(3, 497) = 6.863, *p* < 0.001), with older participants (over 50) showing greater intentions to leave their jobs than younger participants do. These findings indicate that younger employees experience lower levels of job stress and greater job satisfaction, whereas older employees report greater job stress and greater turnover intention.

#### 3.2.3 Monthly income

One-way ANOVA revealed significant differences in job stress, job satisfaction, and turnover intention based on monthly income (see Table 5). Job autonomy stress (*F*(3, 497) = 10.766, *p* < 0.001), workload stress (*F*(3, 497) = 6.658, *p* < 0.001), work relationship stress (*F*(3, 497) = 4.506, *p* < 0.001), and overall job stress (*F*(3, 497) = 7.298, *p* = 0.04) differed significantly across income levels, with lower-income participants (≤TWD 30,000) reporting higher stress levels.

**Table 5:** One-way ANOVA results for monthly income differences in job stress, job satisfaction, and turnover intention.

Dimension	Monthly income (TW$)	*N*	Mean	SD	95% CI lower	95% CI upper	*F*	*p* value	*df*	Scheffé *post hoc* test		
Job autonomy stress	≤30,000	93	2.041	0.504	1.937	2.144	10.766^***^	0.000	(3,497)	1. > 3. > 2.		
	30,001–35,000	191	1.667	0.522	1.593	1.742						
	35,001–50,000	181	1.872	0.581	1.787	1.957						
	>50,000	36	1.772	0.565	1.581	1.963						
	Total	501	1.818	0.559	1.769	1.867	Partial Eta squared = 0.061					
Workload stress	≤30,000	93	2.217	0.678	2.078	2.357	6.658^***^	0.000	(3,497)	1. > 2.		
	30,001–35,000	191	1.870	0.639	1.779	1.961						
	35,001–50,000	181	2.064	0.703	1.961	2.167						
	>50,000	36	1.878	0.586	1.679	2.076						
	Total	501	2.005	0.678	1.946	2.065	Partial Eta squared = 0.039					
Works relationships stress	≤30,000	93	2.312	0.884	2.130	2.494	4.506^**^	0.004	(3,497)	1. > 2.		
	30,001–35,000	191	1.969	0.820	1.852	2.086						
	35,001–50,000	181	2.166	0.898	2.034	2.297						
	>50,000	36	1.889	0.708	1.649	2.129						
	Total	501	2.098	0.863	2.022	2.174	Partial Eta squared = 0.026					
The overall average of job stress	≤30,000	93	2.185	0.653	2.051	2.320	7.298^**^	0.000	(3,497)	1. > 3. > 2.		
	30,001–35,000	191	1.829	0.624	1.740	1.918						
	35,001–50,000	181	2.029	0.694	1.927	2.130						
	>50,000	36	1.841	0.572	1.647	2.034						
	Total	501	1.968	0.664	1.910	2.026	Partial Eta squared = 0.042					
Intrinsic job satisfaction	≤30,000	93	3.464	0.320	3.398	3.530	5.075^**^	0.002	(3,497)	2. > 1.		
	30,001–35,000	191	3.605	0.295	3.563	3.647						
	35,001–50,000	181	3.552	0.300	3.509	3.596						
	>50,000	36	3.618	0.268	3.527	3.709						
	Total	501	3.561	0.303	3.534	3.587	Partial Eta squared = 0.030					
Extrinsic job satisfaction	≤30,000	93	3.816	0.362	3.741	3.890	10.275^***^	0.000	(3,497)	2. > 4. > 3. > 1.		
	30,001–35,000	191	4.079	0.387	4.023	4.134						
	35,001–50,000	181	3.963	0.394	3.905	4.021						
	>50,000	36	4.059	0.412	3.919	4.199						
	Total	501	3.987	0.398	3.952	4.021	Partial Eta squared = 0.0580					
The overall average of job satisfaction	≤30,000	93	3.605	0.288	3.545	3.664	9.276^***^	0.000	(3,497)	4. > 2. > 3. > 1.		
	30,001–35,000	191	3.794	0.292	3.753	3.836						
	35,001–50,000	181	3.717	0.301	3.672	3.761						
	>50,000	36	3.795	0.299	3.693	3.896						
	Total (23 items)	501	3.731	0.303	3.704	3.758	Partial Eta squared = 0.053					
The overall average of turnover intention	≤30,000	93	2.237	0.772	2.078	2.396	4.321^**^	0.005	(3,497)	1. > 2.		
	30,001–35,000	191	1.927	0.736	1.822	2.032						
	35,001–50,000	181	2.099	0.790	1.984	2.215						
	>50,000	36	1.889	0.708	1.649	2.129						
	Total	501	2.044	0.768	1.976	2.111	Partial Eta squared = 0.025					

*N* = 501; **p < 0.0; ***p < 0.001.

With respect to job satisfaction, significant income differences were found for both intrinsic (*F*(3, 497) = 5.075, *p* = 0.002) (*F*(3, 497) = 5.075, *p* = 0.002) (*F*(3, 497) = 5.075, *p* = 0.002) and extrinsic job satisfaction (*F*(3, 497) = 10.275, *p* < 0.001) (*F*(3, 497) = 10.275, *p* < 0.001) (*F*(3, 497) = 10.275, *p* < 0.001). Higher income groups reported significantly greater job satisfaction. The mean score for intrinsic job satisfaction was highest among those earning more than 50,000 TWD (M = 3.618, SD = 0.268), whereas the lowest income group reported the lowest satisfaction levels (M = 3.464, SD = 0.352).

Finally, significant differences were observed in overall turnover intention (*F*(3, 497) = 4.321, *p* = 0.005) (*F*(3, 497) = 4.321, *p* = 0.005) (*F*(3, 497) = 4.321, *p* = 0.005), with higher turnover intention reported by the lowest income group. The mean turnover intention score for this group was 2.172 (SD = 0.736), which was significantly higher than that for those earning more than 50,000 TWD.

These findings suggest that lower-income employees experience greater job stress and turnover intention, whereas higher-income employees report greater job satisfaction. This finding supports Hypothesis 1 (H_1_), indicating that monthly income significantly influences job stress, job satisfaction, and turnover intention among employees.

#### 3.2.4 Education level

One-way ANOVA revealed significant differences in job stress, job satisfaction, and turnover intention by education level (see Table 6). Significant differences were found in job autonomy stress (*F*(2, 498) = 4.163, *p* = 0.010), workload stress (*F*(2, 498) = 3.379, *p* = 0.035), and overall job stress (*F*(2, 498) = 3.705, *p* = 0.025), with lower stress reported by those with a university or college degree than by those with a high school education or below. However, no significant differences were observed in work relationship stress (*F*(2, 498) = 2.863, *p* = 0.058).

**Table 6:** One-way ANOVA results for education level differences in job stress, job satisfaction, and turnover intention.

Dimension	Education level	*N*	Mean	SD	95% CI lower	95% CI upper	*F*	*p* value	*df*	Scheffé *post hoc* test
Job autonomy stress	High school or below	66	1.992	0.578	1.849	2.134	4.613^*^	0.010	(2,498)	1. > 2.
	College	238	1.759	0.568	1.687	1.832				
	Master’s or above	197	1.831	0.532	1.756	1.906				
	Total	501	1.818	0.559	1.769	1.867	Partial Eta squared = 0.018			
Workload stress	High school or below	66	2.203	0.721	2.026	2.380	3.379^*^	0.035	(2,498)	1. > 2.
	College	238	1.961	0.686	1.874	2.049				
	Master’s or above	197	1.992	0.644	1.901	2.082				
	Total	501	2.005	0.678	1.946	2.065	Partial Eta squared = 0.013			
Works relationships stress	High school or below	66	2.333	0.883	2.116	2.550	2.863	0.058	(2,498)	
	College	238	2.067	0.869	1.956	2.178				
	Master’s or above	197	2.056	0.840	1.938	2.174				
	Total	501	2.098	0.863	2.022	2.174	Partial Eta squared = 0.011			
The overall average of job stress	High school or below	66	2.171	0.697	2.000	2.343	3.705^*^	0.025	(2,498)	1. > 2.
	College	238	1.924	0.675	1.837	2.010				
	Master’s or above	197	1.954	0.630	1.865	2.042				
	Total	501	1.968	0.664	1.910	2.026	Partial Eta squared = 0.015			
Intrinsic job satisfaction	High school or below	66	3.428	0.336	3.346	3.511	7.606^**^	0.001	(2,498)	3. > 2. > 1.
	College	238	3.574	0.308	3.535	3.613				
	Master’s or above	197	3.589	0.276	3.551	3.628				
	Total	501	3.561	0.303	3.534	3.587	Partial Eta squared = 0.030			
Extrinsic job satisfaction	High school or below	66	3.831	0.386	3.736	3.926	5.924^**^	0.003	(2,498)	2. > 3. > 1.
	College	238	4.014	0.429	3.959	4.069				
	Master’s or above	197	4.005	0.349	3.956	4.054				
	Total	501	3.987	0.398	3.952	4.021	Partial Eta squared = 0.023			
The overall average of job satisfaction	High school or below	66	3.589	0.313	3.512	3.666	8.603^***^	0.000	(2,498)	3. > 2. > 1.
	College	238	3.750	0.324	3.709	3.791				
	Master’s or above	197	3.756	0.258	3.719	3.792				
	Total (23 items)	501	3.731	0.303	3.704	3.758	Partial Eta squared = 0.033			
The overall average of turnover intention	High school or below	66	2.258	0.771	2.068	2.447	2.964	0.053	(2,498)	
	College	238	2.013	0.771	1.914	2.111				
	Master’s or above	197	2.010	0.756	1.904	2.116				
	Total	501	2.044	0.768	1.976	2.111	Partial Eta squared = 0.012			

*N* = 501; ^*^p < 0.05 ^**^p < 0.01; ^***^p < 0.001 Sig. (2-tailed).

In terms of job satisfaction, both intrinsic (*F*(2, 498) = 13.087, *p* < 0.001) and extrinsic job satisfaction (*F*(2, 498) = 9.045, *p* < 0.001), as well as overall job satisfaction (*F*(2, 498) = 12.494, *p* < 0.001), were higher among those with more advanced education. Turnover intention, however, did not significantly differ by education level (*F*(2, 498) = 2.964, *p* = 0.053). These findings suggest that higher education, particularly at the master’s degree or above, is associated with lower job stress and higher job satisfaction. Individuals with advanced degrees reported less job autonomy stress and greater intrinsic and extrinsic satisfaction. However, education level did not significantly impact work relationship stress or turnover intention, highlighting the role of higher education in enhancing job satisfaction and reducing stress, whereas its influence on turnover intention appears limited.

#### 3.2.5 Job position

Independent samples t tests revealed no significant differences between supervisory and nonsupervisory positions in terms of job stress, job satisfaction, or turnover intention (see Table 7). Specifically, there were no significant differences in job autonomy stress (*t*(499) = 0.550, *p* = 0.583), workload stress (*t*(499) = 0.282, *p* = 0.778), interpersonal relationship stress (*t*(499) = 0.107, *p* = 0.914), or overall job stress (*t*(499) = 0.298, *p* = 0.766).

**Table 7:** Independent samples *t* test results for job position differences in job stress, job satisfaction, and turnover intention.

Dimension	Position level	*N*	Mean	SD	*t*	*df*	Sig. (2-tailed)	95% CI lower	95% CI upper	Cohen’s d
Job autonomy stress	Nonsupervisory	286	1.830	0.561	0.550	499	0.583	−0.072	0.127	0.050
	Supervisory	215	1.802	0.559						
Workload stress	Nonsupervisory	286	2.013	0.686	0.282	499	0.778	−0.103	0.138	0.027
	Supervisory	215	1.995	0.668						
Interpersonal relationships stress	Nonsupervisory	286	2.101	0.879	0.107	499	0.914	−0.145	0.162	−0.095
	Supervisory	215	2.093	0.843						
The overall average of job stress	Nonsupervisory	286	1.976	0.672	0.298	499	0.766	−0.100	0.136	0.027
	Supervisory	215	1.958	0.655						
Intrinsic job satisfaction	Nonsupervisory	286	3.557	0.301	−0.282	499	0.778	−0.062	0.046	−0.026
	Supervisory	215	3.565	0.307						
Extrinsic job satisfaction	Nonsupervisory	286	3.981	0.380	−0.345	499	0.730	−0.083	0.058	−0.015
	Supervisory	215	3.994	0.421						
The overall average of job satisfaction	Nonsupervisory	286	3.727	0.291	−0.679	499	0.726	−0.063	0.044	−0.033
	Supervisory	215	3.737	0.318						
The overall average of turnover intention	Nonsupervisory	286	2.049	0.789	0.169	499	0.866	−0.125	0.148	0.016
	Supervisory	215	2.037	0.742						

*N = 501.*

Similarly, no significant differences were observed in intrinsic job satisfaction (*t*(499) = −0.282, *p* = 0.778), extrinsic job satisfaction (*t*(499) = −0.345, *p* = 0.730), or overall job satisfaction (*t*(499) = −0.679, *p* = 0.726). Turnover intention also did not differ significantly between supervisory and nonsupervisory positions (*t*(499) = 0.169, *p* = 0.866). These results indicate that there are no significant differences between supervisory and nonsupervisory positions in terms of job stress, job satisfaction, or turnover intention.

#### 3.2.6 Marital status

Independent samples t tests revealed no significant differences between married and single participants in terms of job stress, job satisfaction, or turnover intention (see Table 8). Specifically, there were no significant differences in job autonomy stress (*t*(499) = −1.134, *p* = 0.257), workload stress (*t*(499) = −0.562, *p* = 0.574), interpersonal relationship stress (*t*(499) = −0.165, *p* = 0.869), or overall job stress (*t*(499) = −0.528, *p* = 0.561).

**Table 8:** Independent samples *t* test results for marital status differences in job stress, job satisfaction, and turnover intention.

Dimension	Marital status	*N*	Mean	SD	*t*	*df*	Sig. (2-tailed)	95% CI lower	95% CI upper	Cohen’s d
Job autonomy stress	Married	116	1.766	0.566	−1.134	499	0.257	−0.184	0.049	−0.120
	Single	385	1.833	0.557						
Workload stress	Married	116	1.974	0.686	−0.562	499	0.574	−0.182	0.101	−0.060
	Single	385	2.015	0.676						
Interpersonal relationships stress	Married	116	2.086	0.860	−0.165	499	0.869	−0.195	0.165	−0.017
	Single	385	2.101	0.865						
The overall average of job stress	Married	116	1.937	0.671	−0.582	499	0.561	−0.179	0.097	−0.062
	Single	385	1.978	0.663						
Intrinsic job satisfaction	Married	116	3.588	0.314	1.091	499	0.141	−0.026	0.182	0.115
	Single	385	3.553	0.300						
Extrinsic job satisfaction	Married	116	4.031	0.424	1.384	499	0.276	−0.028	0.098	0.146
	Single	385	3.973	0.389						
The overall average of job satisfaction	Married	116	3.765	0.321	−0.151	499	0.167	−0.024	0.141	0.146
	Single	385	3.721	0.296						
The overall average of turnover intention	Married	116	2.034	0.768	−0.151	499	0.880	−0.019	0.107	−0.017
	Single	385	2.047	0.769						

*N = 501.*

Similarly, no significant differences were found in intrinsic job satisfaction (*t*(499) = 1.091, *p* = 0.141), extrinsic job satisfaction (*t*(499) = 1.384, *p* = 0.276), or overall job satisfaction (*t*(499) = −0.151, *p* = 0.167). Turnover intention also did not differ significantly between married and single participants (*t*(499) = −0.151, *p* = 0.880). These findings indicate that marital status does not significantly affect job stress, job satisfaction, or turnover intention.

#### 3.2.7 Dependents’ status

Table 9 presents the independent samples *t* test results comparing job stress, job satisfaction, and turnover intention between employees with and without dependents. The findings indicate that employees with dependents reported significantly greater levels of job autonomy stress (*M* = 1.864 vs. *M* = 2.752), workload stress (*M* = 2.049 vs. *M* = 1.849), interpersonal stress (*M* = 2.148 vs. *M* = 1.918), and overall job stress (*M* = 2.014 vs. *M* = 1.805) than did those without dependents.

**Table 9:** Independent samples *t* test results for dependent status differences in job stress, job satisfaction, and turnover intention.

Dimension	Dependent status-dependent family members	*N*	Mean	SD	*t*	*df*	Sig. (2-tailed)	95% CI lower	95% CI upper	Cohen’s d
Job autonomy stress	Yes	391	1.860	0.576	3.240^**^	499	0.001	0.076	0.311	0.348
	No	110	1.667	0.469						
Workload stress	Yes	391	2.049	0.699	2.752^**^	499	0.006	0.057	0.343	0.297
	No	110	1.849	0.573						
Interpersonal relationships stress	Yes	391	2.148	0.891	2.484^*^	499	0.013	0.048	0.412	0.268
	No	110	1.918	0.731						
The overall average of job stress	Yes	391	2.014	0.687	2.943^**^	499	0.003	0.070	0.349	0.317
	No	110	1.805	0.549						
Intrinsic job satisfaction	Yes	391	3.546	0.311	−2.018^*^	499	0.044	−0.130	−0.002	−0.219
	No	110	3.612	0.268						
Extrinsic job satisfaction	Yes	391	3.958	0.405	−3.074^**^	499	0.002	−0.214	−0.047	−0.332
	No	110	4.089	0.354						
The overall average of job satisfaction	Yes	391	3.711	0.311	−2.833^**^	499	0.005	−0.156	−0.028	−0.306
	No	110	3.803	0.259						
The overall average turnover intention	Yes	391	2.087	0.789	2.376^*^	499	0.018	0.034	0.358	0.257
	No	110	1.891	0.668						

*N* = 501; *p < 0.05; **p < 0.01.

Additionally, employees with dependents had significantly lower intrinsic (*M* = 3.546 vs. *M* = 3.612), extrinsic (*M* = 3.958 vs. *M* = 4.089), and overall job satisfaction (*M* = 3.711 vs. *M* = 3.803). Turnover intention was also greater among employees with dependents (*M* = 2.087 vs. *M* = 1.891). These results suggest that employees with dependents experience greater job stress, lower job satisfaction, and greater turnover intention than do those without dependents.

### 3.3 Results of the correlation matrix

Table 10 presents the Pearson correlation coefficients among the dimensions of job stress, job satisfaction, and turnover intention. The analysis reveals several significant correlations at the *p* < 0.001 level. Among the job stress dimensions, strong positive correlations were observed between workload stress and interpersonal relationship stress (*r* = 0.930) and between job autonomy stress and the overall average level of job stress (*r* = 0.897).

**Table 10:** Correlation matrix for job stress, job satisfaction, and turnover intention.

Dimension	1	2	3	4	5	6	7	8
Job autonomy stress	1							
Workload stress	0.868^***^	1						
Interpersonal relationships stress	0.738^***^	0.930^***^	1					
The overall average of job stress	0.897^***^	0.981^***^	0.958^***^	1				
Intrinsic job satisfaction	−0.559^***^	−0.496^***^	−0.407^***^	0.501^***^	1			
Extrinsic job satisfaction	−0.701^***^	−0.588^***^	−0.462^***^	0.596^***^	0.572^***^	1		
The overall average of job satisfaction	−0.705^***^	−0.607^***^	−0.488^***^	0.615^***^	0.902^***^	0.870^***^	1	
The overall average turnover intention	0.704^***^	0.901^***^	0.968^***^	0.924^***^	−0.441^***^	−0.468^***^	−0.511^***^	1

*N* = 501; ***p < 0.001; Sig. (2-tailed).

The strong positive correlation between workload stress and interpersonal relationships stress (*r* = 0.930) indicates that as employees experience more workload stress, they tend to also experience higher levels of interpersonal relationships stress. These two types of stress tend to increase together. In other words, an increase in workload may lead to more challenging or strained interpersonal relationships at work.

Similarly, the strong positive correlation between job autonomy stress and the overall average of job stress (*r* = 0.897) suggests that when employees experience greater job autonomy stress, their overall level of job stress also tends to be higher. This implies that the less autonomy employees have in their work, the higher their overall perceived job stress becomes. These strong positive correlations demonstrate that different types of job stress are interrelated. When stress in a dimension increases, stress in other dimensions often rises as well, further emphasizing the multidimensional impact of stress in the workplace.

Additionally, the overall average job stress is strongly negatively correlated with both intrinsic job satisfaction (*r* = −0.705) and extrinsic job satisfaction (*r* = −0.607). These findings indicate that higher levels of job stress are consistently associated with lower job satisfaction. Moreover, turnover intention is positively correlated with the overall average level of job stress (*r* = 0.704) and negatively correlated with both intrinsic (*r* = −0.441) and extrinsic job satisfaction (*r* = −0.511), suggesting that employees experiencing greater job stress and lower job satisfaction are more likely to have greater turnover intentions. These results present the interconnectedness of job stress, job satisfaction, and turnover intention, highlighting the importance of addressing stress factors in the workplace to increase job satisfaction and reduce turnover intention.

### 3.4 Results of regression analysis

#### 3.4.1 Regression analysis results for predicting intrinsic job satisfaction

Table 11 shows that job autonomy stress significantly predicts intrinsic job satisfaction (*F* = 227.217, *p* < 0.001), explaining 31.3% of the variance (*R*^2^ = 0.313). The Durbin–Watson statistic of 1.847 indicates no autocorrelation. Moreover, Table 11 shows the coefficients, with job autonomy stress having a significant negative effect on intrinsic job satisfaction (*β* = −0.559, *p* < 0.001). The unstandardized coefficient is −0.303, with a standard error of 0.020. The VIF of 1 confirms that there is no multicollinearity. These results support H₃, indicating that job autonomy stress is a significant predictor of intrinsic job satisfaction.

**Table 11:** Regression analysis summary for predicting intrinsic job satisfaction.

Model		Unstandardized coefficients		Standardized coefficients	*t*	Sig. (2-tailed)	95% CI lower	95% CI upper	Collinearity statistics	
		*Β* (constant)	Std. error	*β*					Tolerance	VIF
1	(Constant)	4.112	0.038		107.457^***^	0.000	4.037	4.187		
	Job autonomy stress	−0.303	0.020	−0.559	−15.074^***^	0.000	−0.343	−0.264	1	1
*R* = 0.559										
*R^2^* Square = 0.313										
Adjusted *R^2^* = 0.312										
*df* = (1, 499)										
*F* = 227.217^***^ (*p* = 0.000)										
Durbin-Watson Test = 1.847										

Dependent variable: the intrinsic job satisfaction.

*N* = 501; ^***^p < 0.001.

#### 3.4.2 Regression analysis results for predicting extrinsic job satisfaction

Table 12 shows that job autonomy stress significantly predicts extrinsic job satisfaction (*F* = 481.561, *p* < 0.001), explaining 49.1% of the variance (*R*^2^ = 0.491). Adding interpersonal relationship stress to Model 2 slightly improved the model, increasing the explained variance to 49.8% (*R*^2^ = 0.498, *F* = 246.896, *p* < 0.001). Moreover, Table 12 indicates that job autonomy stress has a strong negative effect on extrinsic job satisfaction (*β* = −0.701, *p* < 0.001), whereas interpersonal relationship stress has a small but significant positive effect (*β* = 0.122, *p* < 0.05). No multicollinearity issues were detected. These results confirm that both job autonomy stress and interpersonal relationship stress are significant predictors of extrinsic job satisfaction (H_3_).

**Table 12:** Regression analysis summary for predicting extrinsic job satisfaction.

Model		Unstandardized coefficients		Standardized coefficients	*t*	Sig. (2-tailed)	95% CI lower	95% CI upper	Collinearity statistics	
		*Β* (constant)	Std. error	*β*					Tolerance	VIF
1 (a)	(Constant)	4.892	0.043		113.317^***^	0.000	4.807	4.977		
	Job autonomy stress	−0.498	0.023	−0.701	−21.944^***^	0.000	−0.543	−0.454	1	1
2 (b)	(Constant)	4.890	0.043		113.915^***^	0.000	4.806	4.975		
	Job autonomy stress	−0.562	0.033	−0.791	−16.802^***^	0.000	−0.628	−0.496	0.455	2.197
	Interpersonal relationships stress	0.056	0.022	0.122	2.591^*^	0.010	0.014	0.099	0.455	2.197
Model 1 (a)					Model 2 (b)					
*R* = 0.701					*R* = 0.706					
*R^2^* = 0.491					*R*^2^ = 0.498					
Adjusted *R*^2^ = 0.490					Adjusted *R*^2^ = 0.496					
*df* = (1, 499)					*df* = (2, 498)					
*F* = 481.561^***^ (*p* = 0.000)					*F* = 246.896^***^ (*p* = 0.000)					
					Durbin-Watson Test = 1.587					

Dependent variable: the extrinsic job satisfaction.

*N* = 501; ^*^p < 0.05; ^***^p < 0.001.

#### 3.4.3 Regression analysis results for predicting the overall average turnover intention

Table 13 presents the regression analysis model summary and ANOVA results for predicting the overall average turnover intention (H₄). Model 1, which includes interpersonal relationship stress as the sole predictor, explains 93.7% of the variance (*R*^2^ = 0.937, *F* = 7477.020, *p* < 0.001). As additional predictors are included in Models 2 through 4, the explained variance slightly increases, with Model 4 achieving an *R*^2^ of 0.942 (*F* = 2022.673, *p* < 0.001).

**Table 13:** Regression analysis summary for predicting the overall average turnover intention.

Model		Unstandardized coefficients		Standardized coefficients	*t*	Sig.	95% CI lower	95% CI upper	Collinearity statistics	
		*Β* (constant)	Std. error	*β*					Tolerance	VIF
1	(Constant)	0.236	0.023		10.419^***^	0.000	0.191	0.280		
	Interpersonal relationships stress	0.862	0.010	0.968	86.470^***^	0.000	0.842	0.882	1.000	1.000
2	(Constant)	0.786	0.120		6.574^***^	0.000	0.551	1.020		
	Interpersonal relationships stress	0.842	0.011	0.945	78.701^***^	0.000	0.821	0.863	0.834	1.199
	Intrinsic job satisfaction	−0.142	0.030	−0.056	−4.684^***^	0.000	−0.202	−0.083	0.834	1.199
3	(Constant)	1.061	0.139		7.639^***^	0.000	0.788	1.334		
	Interpersonal relationships stress	0.878	0.014	0.986	61.427^***^	0.000	0.850	0.906	0.455	2.197
	Intrinsic job satisfaction	−0.195	0.033	−0.077	−5.883^***^	0.000	−0.260	−0.130	0.687	1.455
	Job autonomy stress	−0.091	0.024	−0.066	−3.756^***^	0.000	−0.139	−0.043	0.375	2.667
4	(Constant)	1.286	0.175		7.329^***^	0.000	0.941	1.630		
	Interpersonal relationships stress	0.881	0.014	0.990	61.449^***^	0.000	0.853	0.909	0.449	2.228
	Intrinsic job satisfaction	−0.173	0.035	−0.068	−4.987^***^	0.000	−0.241	−0.105	0.623	1.605
	Job autonomy stress	−0.121	0.028	−0.088	−4.305^***^	0.000	−0.176	−0.066	0.280	3.574
	Extrinsic job satisfaction	−0.064	0.031	−0.033	−2.083^*^	0.038	−0.125	−0.004	0.455	2.196
Model 1 (a)			Model 2 (b)		Model 3 (c)			Model 4 (d)		
*R* = 0.968			*R* = 0.970		*R* = 0.970			*R* = 0.971		
*R*^2^ = 0.937			*R*^2^ = 0.940		*R*^2^ = 0.942			*R*^2^ = 0.942		
Adjusted *R*^2^ = 0.937			Adjusted *R*^2^ = 0.939		Adjusted *R*^2^ = 0.491			Adjusted *R*^2^ = 0.942		
*df* = (1, 499)			*df* = (2, 498)		*df* = (3, 497)			*df* = (4, 496)		
*F* = 7477.020^***^ (*p* = 0.000)			*F* = 3906.335^***^ (*p* = 0.000)		*F* = 2677.456^***^ (*p* = 0.000)			*F* = 2022.673^***^ (*p* = 0.000)		
								Durbin-Watson Test = 2.293		

Dependent variable: the overall average turnover intention.

*N* = 501; ^*^p < 0.05; ^***^p < 0.001.

Moreover, Table 13 shows that interpersonal relationship stress is the strongest positive predictor of turnover intention across all the models (β = 0.990 in Model 4, *p* < 0.001). Intrinsic job satisfaction, job autonomy stress, and extrinsic job satisfaction have significant negative effects on turnover intention, with β values of −0.068, −0.088, and −0.033, respectively (all *p* < 0.05). The variance inflation factor (VIF) values indicate no serious multicollinearity issues. These results confirm that interpersonal relationship stress is a dominant predictor of turnover intention, with job satisfaction and job autonomy stress also playing significant roles (H_4_).

## 4 Discussions

This study aimed to investigate the relationships among job stress, job satisfaction, and turnover intentions among bank employees in central Taiwan during the COVID-19 pandemic, with a focus on demographic differences. The findings provide several significant insights into how the pandemic has shaped these relationships within the banking sector, and they also resonate with or diverge from previous research.

### 4.1 Demographic differences in job stress, job satisfaction, and turnover intention

The analysis revealed that age, income, education level, and dependent status significantly influenced job stress, job satisfaction, and turnover intention during the COVID-19 pandemic. This finding supports Hypothesis 1, confirming that certain demographic variables exhibit significant differences.

The results of this study show that younger employees reported lower levels of job stress and higher job satisfaction than their older counterparts did. This finding suggests that younger workers may have perceived the global economic downturn as limiting better job opportunities and demonstrated greater adaptability to the rapidly changing work environments brought about by the pandemic. However, these results contrast with those reported by Peter et al. (2024), who reported that younger healthcare professionals exhibited a greater intention to leave both their organizations and the profession prematurely across various work settings. Similarly, Liu et al. (2019) reported that younger rural health workers (under 41 years old), earning a monthly income of $326.8–$490.1 and working in township hospitals, were more likely to express turnover intentions when experiencing low job satisfaction. The discrepancies between these findings and those of the current study may be attributable to differences in the industries and contexts represented within the study samples.

Furthermore, the increased levels of stress and turnover intentions observed among older employees during the pandemic underscore the specific vulnerabilities of this demographic in crisis contexts. Specifically, older employees experienced heightened stress levels and exhibited a greater tendency to consider leaving their jobs during the pandemic. These findings emphasize the need to address the specific needs and challenges of older workers when navigating crisis scenarios, suggesting that targeted interventions may be needed to support this demographic effectively.

The present study revealed that monthly income significantly influences employees’ job stress, job satisfaction, and turnover intention, which aligns with findings from previous studies (Liu et al., 2019; Yan et al., 2021). Specifically, employees with lower income (<30,000 TWD) presented higher levels of job stress and turnover intention and lower job satisfaction, which may reflect the increased economic pressure and job insecurity they face. This outcome supports stress theory, suggesting that financial hardship can exacerbate psychological stress and job dissatisfaction, ultimately leading to a greater likelihood of turnover (Hur, 2024). Conversely, the study revealed that higher-income groups reported significantly greater job satisfaction, which may be attributed to the financial security and resources provided by higher earnings. This finding is consistent with prior research, which underscores the role of economic compensation in enhancing job satisfaction and reducing turnover intention (Hur, 2024; Yan et al., 2021).

Moreover, the significant differences observed in job autonomy stress, workload stress, and work relationship stress across income levels further highlight the critical role that economic factors play in the work environment. These findings suggest that to improve overall job satisfaction and reduce turnover rates, organizations should consider revising their compensation structures, particularly by providing additional support to lower-income employees to alleviate their work-related stress. This study underscores the importance of monthly income as a key variable influencing job stress, job satisfaction, and turnover intention. Future research could further explore how other socioeconomic factors, such as career advancement opportunities and professional development, impact employee attitudes and behaviors across different income levels.

In this study, educational attainment did not significantly affect turnover intention during the COVID-19 pandemic. Although this finding contrasts with the results reported by Federico et al., McCloskey, and Braito and Caston, who reported that higher educational attainment was associated with increased turnover rates (Chen et al., 2022), the results of this study suggest that other factors, such as workplace support and resilience, may have played a more critical role in influencing turnover intention during this period.

The finding that job position (i.e., supervisory vs. nonsupervisory roles) did not significantly impact job stress, job satisfaction, or turnover intention is somewhat surprising, particularly compared with studies that indicated a higher job position, higher job satisfaction and lower or higher turnover intention among supervisors by Yin and Ana and Weisman (Chen et al., 2022). This discrepancy could be attributed to the unique context of the banking sector during the pandemic, where stressors may have been more evenly distributed across different job roles.

Moreover, the effects of marital status and gender on job stress, job satisfaction, and turnover intention were not statistically significant in this study. These findings differ from those of previous research by Mobley, Marsh, and Mannari, as cited by Chen et al. (2022). However, this study revealed that employees with dependents faced significantly greater challenges during the COVID-19 pandemic. This variation suggests that the relationship between these demographic factors and work-related outcomes may be more complex and context-dependent than previously assumed. Nonetheless, the need for organizational policies that support work–life balance, particularly during crises, remains crucial.

### 4.2 Correlation and regression analysis of job stress, job satisfaction, and turnover intention

The findings from the Pearson correlation analysis reveal significant relationships between various job stress factors, job satisfaction, and turnover intentions among employees. Specifically, job autonomy stress, workload stress, and interpersonal relationship stress were all positively correlated with overall job stress, indicating that increases in these specific stressors correspond with increases in overall job stress. Furthermore, these stressors were negatively correlated with both intrinsic and extrinsic job satisfaction, suggesting that higher levels of job-related stress are associated with lower job satisfaction across different dimensions, which aligns with the literature on occupational stress and its impact on work outcomes (Ahsan et al., 2009; Cummins, 1990; Fletcher and Payne, 1980; Klassen et al., 2009; Koslowsky et al., 1995; Landsbergis, 1988; Stamps and Piedmonte, 1986; Vinokur-Kaplan, 1990; Yang et al., 2018). This finding supports Hypothesis 2, confirming the relationships among job stress factors, job satisfaction, and turnover intentions among employees.

The regression analysis further highlights the pivotal role of job autonomy stress in predicting both intrinsic and extrinsic job satisfaction. The results demonstrate that job autonomy stress significantly predicts intrinsic and extrinsic job satisfaction, emphasizing that a lack of control or autonomy in one’s job significantly diminishes overall job satisfaction. This finding is supported by Deci and Ryan’s (1985) self-determination theory, which emphasizes the importance of autonomy in fostering intrinsic motivation and satisfaction. Additionally, the introduction of interpersonal relationship stress in predicting extrinsic job satisfaction reveals a unique insight: while job autonomy stress negatively impacts satisfaction, strong interpersonal relationships may partially mitigate this effect, particularly in the extrinsic dimension of job satisfaction. This finding is consistent with social support theory, which posits that positive social interactions at work can buffer the negative effects of job stress (House, 1981). When examining turnover intention, the regression models indicate that interpersonal relationship stress is the most substantial predictor, followed by intrinsic job satisfaction and job autonomy stress. These findings align with broader literature suggesting that poor interpersonal relationships in the workplace can significantly increase turnover intentions (Jiang et al., 2022; Li et al., 2022), whereas high levels of job satisfaction, particularly intrinsic satisfaction, can buffer against the desire to leave (Obuobisa-Darko and Sokro, 2023; Takyi et al., 2023; Said and El-Shafei, 2021; Wang and Huang, 2018; Wu et al., 2022; Hong, 2021; Zhang and Ru, 2021; Xu, 2021; Chen and Huang, 2019; Feng et al., 2020; Yang and Lu, 2017; Tsai and Lin, 2018; Su and Zhu, 2018; Hoboubi et al., 2017; Wu, 2017; Wu et al., 2022; Li and Hong, 2022; Lin et al., 2022; Hong, 2021; Zhang and Ru, 2021; Xu, 2021; Xu et al., 2022; Tsai and Lin, 2018; Tsai and Wu, 2022; Su and Zhu, 2018). These findings support Hypotheses 3 and 4.

In conclusion, this study highlights the critical importance of managing job stressors, particularly job autonomy stress and interpersonal relationship stress, to increase job satisfaction and reduce turnover intention. The results emphasize that organizations should focus on improving job autonomy and fostering positive interpersonal relationships in the workplace. These efforts could substantially mitigate the adverse effects of job stress on job satisfaction and turnover, promoting a more stable and satisfactory workforce during challenging times such as the COVID-19 pandemic. Furthermore, future research should continue to explore these dynamics in different organizational contexts to further validate these findings and inform effective stress management interventions.

### 4.3 Practical implications for the banking sector during the pandemic

Although these findings were collected during the COVID-19 pandemic, they provide valuable insights that can guide banks in addressing similar challenges in the future. The results suggest that organizations should prioritize interventions targeting key stressors, such as offering tailored support for older employees, lower-income workers, and those with dependents. This recommendation underscores the importance of customized organizational support in crisis contexts. Moreover, fostering a positive work environment that emphasizes strong interpersonal relationships is crucial, particularly during times of crisis, as evidenced by the significant link between relationship stress and turnover intention identified in this study.

### 4.4 Limitations and future research suggestions

Although this study offers valuable insights, its limitations must be acknowledged. The cross-sectional design limits the ability to draw causal conclusions, and the focus on bank employees in central Taiwan may restrict the generalizability of the findings to other regions or industries. The context of the COVID-19 pandemic likely influenced the results, making it essential for future research to consider longitudinal studies that explore how these dynamics evolve over time, particularly in response to organizational interventions during and after crises. Expanding research to include diverse industries and regions would provide a more comprehensive understanding of these dynamics in various contexts. Future research should consider employing longitudinal designs to better establish causal relationships. Expanding the sample to include bank employees from different regions or countries would increase the generalizability of the results. Additionally, future studies could explore other factors, such as organizational culture and leadership style, which may also influence job stress, job satisfaction, and turnover intention.

In conclusion, this study underscores the critical importance of addressing job stress and turnover intention within the banking sector, particularly considering demographic differences. By implementing targeted strategies to effectively manage job stress and enhance job satisfaction, banks can significantly improve employee retention and overall organizational performance. The findings of this study have substantial theoretical and practical implications. Theoretically, this research deepens the understanding of the interplay between job stress, job satisfaction, and turnover intention in the banking industry, highlighting the influence of demographic factors. Practically, the results indicate that bank management should prioritize improving work relationships and managing job autonomy stress as key strategies to mitigate turnover intentions. Tailored stress management programs and initiatives designed to increase job satisfaction across different demographic groups could be effective approaches to bolster employee retention.

## 5 Conclusion

This study provides valuable insights into the relationships among job stress, job satisfaction, and turnover intentions among bank employees in central Taiwan during the COVID-19 pandemic. The findings reveal that job stress significantly negatively impacts job satisfaction and positively correlates with turnover intention, underscoring the importance of managing workplace stress to maintain employee satisfaction and reduce turnover rates during challenging periods. Demographic factors, including age, income, education level, and dependent status, significantly moderated these relationships. Employees with dependents reported greater job stress and turnover intention and lower job satisfaction, highlighting the need for targeted support to alleviate these pressures. Similarly, younger, higher-income, and more educated employees experienced lower stress and higher satisfaction, suggesting that they may have better coping mechanisms or resources. In contrast, older employees, those with lower incomes, and those with less education might benefit from additional support and resources to help them manage stress and enhance job satisfaction during such crises.

The study also identified interpersonal relationship stress as the strongest predictor of turnover intention, emphasizing the critical role of workplace relationships in employee retention. Enhancing the quality of interpersonal interactions and providing support for positive workplace relationships are effective strategies for reducing turnover, particularly in high-stress environments such as banking during a global crisis. The findings suggest that organizations should prioritize interventions that reduce job stress and enhance job satisfaction, with tailored strategies for older employees, lower-income workers, less educated employees, and those with dependents, who were found to be more vulnerable to high stress and low satisfaction during the pandemic. Offering financial planning resources, continuing education opportunities, and flexible work arrangements could be beneficial for these groups. Whereas this study offers important insights, its cross-sectional design and focus on a specific geographic region limit the generalizability of the findings. Future research should adopt longitudinal approaches to better understand the evolving dynamics of job stress, job satisfaction, and turnover intention over time and consider expanding the research to other sectors and regions.

In conclusion, managing job stress and fostering job satisfaction are crucial for reducing turnover intentions among bank employees, especially during crises such as the COVID-19 pandemic. Organizations should focus on improving interpersonal relationships, supporting vulnerable demographic groups, and providing targeted support for employees with dependents to enhance overall employee well-being and retention.

## Data Availability

The original contributions presented in the study are included in the article/supplementary material, further inquiries can be directed to the corresponding author.
